# Genetic variation of the transcription factor GATA3, not STAT4, is associated with the risk of type 2 diabetes in the Bangladeshi population

**DOI:** 10.1371/journal.pone.0198507

**Published:** 2018-07-25

**Authors:** Nafiul Huda, Md. Ismail Hosen, Tahirah Yasmin, Pankaj Kumar Sarkar, A. K. M. Mahbub Hasan, A. H. M. Nurun Nabi

**Affiliations:** 1 Laboratory of Population Genetics, Department of Biochemistry and Molecular Biology, University of Dhaka, Dhaka, Bangladesh; 2 Dinajpur Diabetes O Swasthoseba Hospital, Dinajpur, Bangladesh; China Medical University, TAIWAN

## Abstract

Type 2 diabetes mellitus is a multifactorial metabolic disorder caused by environmental factors and has a strong association with hereditary issues. These hereditary issues result in an imbalance in CD4^+^T cells and a decreased level of naïve CD4^+^T cells, which may be critical in the pathogenesis of type 2 diabetes. Transcription factors GATA3 and STAT4 mediate the cytokine-induced development of naïve T cells into Th1 or Th2 types. In the present study, genetic analyses of GATA3 SNP rs3824662 and STAT4 SNP rs10181656 were performed to investigate the association of allelic and genotypic variations with the risk of T2D in the Bangladeshi population. A total of 297 unrelated Bangladeshi patients with type 2 diabetes and 247 healthy individuals were included in the study. The allelic and genotypic frequencies of rs10181656 located in the STAT4 gene were not found to be associated with risk of type 2 diabetes. The GATA3 rs3824662 T allele and mutant TT genotype had a significant association with the risk of T2D [OR: 1.52 (1.15–2.02), X^2^ = 8.66, *p* = 0.003 and OR: 2.98 (1.36–6.55), X^2^ = 7.98, *p* = 0.04, respectively]. Thus, the present study postulates that the genetic variation of the transcription factor GATA3, not STAT4, is associated with the risk of type 2 diabetes in the Bangladeshi population.

## Introduction

Diabetes is a multifaceted metabolic disorder caused by impaired glucose metabolism characterized by hyperglycemia and is mainly classified as type 1 mellitus and type 2 diabetes mellitus. Both environmental and hereditary components play pivotal roles in the onset of diabetes [1,2]. Also, risk of type 2 diabetes is higher in certain ethnic groups [3] Impaired glucose metabolism in type 1 diabetes is due to the complete destruction of beta cells, while in the case of type 2 diabetes, this phenomenon arises due to insulin resistance and beta cell dysfunction. Although type 1 diabetes has long been considered an autoimmune disorder, researchers now suggest redefining type 2 diabetes as a disease of the immune system rather than a purely metabolic disorder [4,5]. Based on observations at the genetic level, the candidate loci for type 1 and type 2 diabetes appear to be primarily distinct, and susceptible genes for these diseases and have generally not been shown to overlap [6]. Loss of β cell mass and function is the major phenomena marking the development of both type 1 diabetes and type 2 diabetes. β cell dysfunction-mediated imbalance of insulin sensitivity, followed by the development of insulin resistance, triggers pathogenesis of type 2 diabetes [7]. Thus, as both diseases are due to abnormal β-cell function and destruction, it has been thought that type 1 diabetes and type 2 diabetes may share a degree of common genetic predisposition [8]. Many new loci associated with type 2 diabetes have been revealed through screening of genome-wide association studies [9,10].

T2D has known imbalances of CD4^+^T cells and decreased levels of naïve CD4^+^T cells, which in turn play an important role in the pathogenesis of type 2 diabetes. GATA 3 binding protein (GATA3) and Signal Transducer and Activator of Transcription-4 (STAT4) transcription factors mediate the cytokine-induced development of naïve T cells into Th1 or Th2 type. GATA3 consists of six exons and encodes a transcription factor with two transactivation domains and two zinc finger domains [11], which are essential for early T cell development [12]. Researchers found that GATA3 polymorphism is significantly associated with the susceptibility of pediatric B-lineage acute lymphoblastic leukemia [13–16]. Also, studies have demonstrated the upregulation of Th1 cells in adipose tissue and peripheral blood in prediabetic and type 2 diabetic individuals [17]. While a declining trend of naïve CD4^+^T cells [18] and imbalance of CD4^+^T cell subsets, including Treg, Th1, and Th17 [19], have been observed in patients with type 2 diabetes. GATA3, a transcription factor, is a master regulator of Th2 [20] that controls differentiation of CD4^+^T cells. Expression of GATA3 is important to avoid cell death. The SNP rs3824662 mapping is within intron 3 of the transcription factor and putative tumor suppressor gene GATA3. Genome-wide association studies have reported an association of the GATA3 rs3824662 polymorphism (T allele) with childhood acute lymphoblastic leukemia [9] as well as in adolescents and young adults [21]. Studies with animal models have demonstrated that the disappearance of regulatory T cells is a signature of insulin resistance in obese mice models [22]. In addition, impaired expression of GATA3 is associated with obesity [23]. Mutations in the GATA3 gene are linked with several diseases, such as breast cancer, hematological carcinomas and hypoparathyroidism-deafness-renal (HDR) dysplasia syndrome [21,24]. Signal transducer and activator of transcription 4 (STAT4) transduces signals of cytokines to modulate many innate and adaptive immune responses that may contribute to autoimmune responses [25]. STAT4 rs10181656 has been found to be associated with autoimmune diseases like rheumatoid arthritis and systemic lupus erythematosus [26].

An association of specific alleles of transcription factors with type 2 diabetes has been established in other studies that suggested a modulation of this association through obesity, a pivotal risk factor for type 2 diabetes. The rs7903146C>T single nucleotide polymorphism in the transcription factor seven-like 2 (TCF7L2) gene is the locus most strongly associated with type 2 diabetes in different populations, such as Icelandic individuals [27] and individuals of European descent [28]. Polymorphisms present in other genes that encode for transcription factors such as Foxp3, TBX21, and STAT4 are associated with the risk of type 1 diabetes but not with type 2 diabetes [29,30]. More surprisingly, data from genome-wide association studies have revealed that genes associated with the risk of cancer are also risk factors for type 2 diabetes but not for type 1 diabetes [31]. Considering these findings, the present study was undertaken to investigate the allelic and genotypic variations in type 2 diabetic patients in the Bangladeshi population and thus evaluate the probable risk association with the disease.

## Materials and methods

### Subject selection and sample collection

A total of 544 unrelated Bangladeshi individuals were enrolled in this study after getting their consent. Of the 544 participants, 297 (54.6%) individuals were in the patient group with type 2 diabetes, and 247 (45.4%) were healthy individuals. The study was conducted according to the guidelines approved by the ethical review committee of the Faculty of Biological Sciences, University of Dhaka, Bangladesh. After obtaining their verbal consent, an expert phlebotomist collected five to six milliliters of blood from each individual. Plasma samples were used to analyze fasting plasma glucose, glycated hemoglobin (HbA1C), creatinine and alanine aminotransferase using standard methods. Type 2 diabetic patients were confirmed clinically using the levels of fasting plasma glucose (FPG) and HbA_1_C, according to the criteria set by the World Health Organization (WHO). Healthy individuals included students, university employees, and hospital personnel who did not show clinical features of diabetes or other complications, such as acute or chronic infection or kidney and liver diseases. Pregnant women were also excluded from this study. Anthropometric and demographic data such as gender, height, weight, systolic blood pressure (SBP), diastolic blood pressure (DBP), and family history of diabetes were collected and recorded in a well-defined questionnaire.

### Extraction of genomic DNA and analyses of genotypes

Cellular fractionation of the collected blood was used to extract genomic DNA. The quantity and quality of the extracted DNA were verified according to our previous method [32,33]. Amplification of the target regions of GATA3 and STAT4 for studying rs3824662 and rs10181656 polymorphisms, came from a web-based primer designing tool available at http://bioinfo.ut.ee/primer3-0.4.0 as shown in Table 1. Finally, primer sequences were obtained from IDT (USA).

**Table 1:** Primer sequences for amplifying GATA3 and STAT4 genes to study rs3824662 and rs10181656 polymorphisms.

Gene	Primer sequence	Product size
GATA3	Forward outer primer: 5’-TTGCAAATGGAAGAGGGTCT-3’	691 base pairs
	Reverse outer primer: 5’-ACCCTGCAAATGAGAGGAAA-3’	
	G specific primer: 5’-TGAGATTAAACACAAACACGtTG-3’	506 base pairs
	T specific primer: 5’-CTGAGATTAAACACAAACACGaTT-3’	
STAT4	Forward Primer> 5’-AGTTTTCAAAGTCTAACACTGTG-3’	157 base pairs
	Reverse Primer> 5’-GCTGCCATGTCGAGAGTA-3’	

Allele-specific primers were used for the amplification of the GATA3 gene. G or T allele-specific primer is very sensitive and binds only to the DNA fragments containing either G or T nucleotide template DNA. Forward outer and reverse outer primers were designed to amplify a specific 691 base pair region of GATA3. This 691 base pair region contains the desired polymorphic site of interest. Allele-specific primers produce 506 bp products upon the presence of either a G allele or T allele. Briefly, PCR was performed in a total volume of 15 μL with an initial denaturing step of 5 min at 95°C, followed by 40 cycles of 30 s at 95°C, 45 s at 55°C, and 1 min at 72°C, and a final extension step of 5 min at 72°C. Primer concentration used was 200 nM each. The PCR products were separated on an ethidium bromide-stained 2.5% agarose gel, visualized with UV light, and photographed. This protocol has been deposited in protocols.io that has been assigned a DOI number http://dx.doi.org/10.17504/protocols.io.k45cyy6.

Upon PCR using specific primers (presented in Table 1) for the STAT4 gene, a 157 bp amplicon that contained the desired polymorphic site for rs10181656 was found. After gel electrophoresis, the PCR products were stored at 4°C and subjected to restriction digestion to analyze whether the polymorphic site contains either a G or C allele. G is the ancestral allele, while C is the mutant allele. Similarly, PCR was performed in a total volume of 15 μL with an initial denaturing step of 5 min at 95°C. Again, followed by 40 cycles of 30 s at 95°C, 45 s at 55°C, and 1 min at 72°C, and a final extension step of 5 minutes at 72°C. The PCR products were separated on an ethidium bromide-stained 2.5% agarose gel, visualized with UV light, and photographed. After performing polymerase chain reaction to determine the presence of the rs10181656 polymorphism in the STAT4 gene, 157 base pair PCR products were digested with the *Dde*I (C’TNAG) (Thermo Fisher Scientific, USA) restriction enzyme according to the manufacturer’s instructions and run in a 2.5% agarose gel electrophoresis stained with ethidium bromide. The *Dde*I cleaved the 157 base pair PCR products into two fragments of 102 base pairs and 55 base pairs when the G allele was present (GG), whereas the C allele was not digested and displayed only the 157 base pair band (CC). In the case of heterozygous GC, 157 base pair, 102 base pair and 55 base pair bands were found. The digestion took 1 hour at 37°C for completion.

### Statistical analyses

The results were expressed as the mean±SD for continuous variables and % for categorical variables. Independent Student’s t-tests were performed to compare the differences between variables from the control and diabetic patients.

## Results

### Demographic, anthropometric and clinical data of study participants

Among the 297 type 2 diabetes patients, 170 (57.23%) were female, and 127 (42.77%) were male. The average BMI of individuals with type 2 diabetes was 23.95±2.85, and the average age was 48.59±9.72 years. The mean systolic blood pressure of type 2 diabetes subjects was 121.73±6.01 mmHg, while the mean diastolic blood pressure was 82.02±6.05 mmHg. Out of 247 healthy controls, 144 (58.3%) were female, and 103 (41.7%) were male. The average BMI of the control individuals was 23.05±4.59, and the average age was 43.79±13.1. The mean systolic and diastolic blood pressure of the controls was 124.11±11.32 and 73.80±5.85 mmHg, respectively. The anthropometric and demographic data for male and female type 2 diabetes patients and healthy individuals are presented in Table 2.

**Table 2:** The demographic, anthropometric and clinical characteristics of Type 2 diabetic patients and controls.

Parameters	Type 2 diabetic patients		Healthy individuals	
	n = 297		n = 247	
	Female	Male	Female	Male
Age (years)	48.34±9.26	48.45±8.95	42.56±13.48	45.52±13.1
Height (cm)	159±6.70	159±7.32	160.40±8.89	168.18±6.22
Weight (kg)	60.33±8.57	60.85±6.11	59.17±11.50	64.39±9.57
BMI (kg/m^2^)	23.94±2.09	24.14±3.12	23.21±5.15	22.84±3.71
SBP (mmHg)	121 ± 6.5	123± 7.0	123.0 ± 14.0	125.17 ± 5.82
DBP (mmHg)	82.0 ± 6.50	81.0 ± 3.10	72 ± 5.11	76.30 ± 5.91
FPG (mmol/L)	8.85 ± 3.97*	9.15 ± 2.73^#^	4.28 ± 1.30*	4.26 ± 1.06^#^
HbA1C (%)	9.95 ± 3.15*	10.08 ± 1.80^#^	4.43 ± 1.08*	4.60 ± 1.05^#^
Creatinine (mg/dL)	1.12 ± 0.40	1.17 ± 1.01	0.94 ± 0.29	0.95 ± 0.24
ALT (IU/L)	42.73 ± 3.94*	43.48±3.58^#^	37.26 ± 2.92*	35.07 ± 2.89^#^

BMI: Body mass index; SBP: Systolic blood pressure; DBP: Diastolic blood pressure; FPG: Fasting plasma glucose; HbA1C: Glycated hemoglobin; ALT: Alanine transaminase.

*p<0.05 (Female vs Female between the study groups)

^#^p<0.05 (Male vs Male between the study groups).

The mean level of fasting plasma glucose in the study participants with type 2 diabetes was 9.14±1.10 mmol/L, while this value in healthy individuals was measured to be 4.27±1.20 mmol/L. The estimated value of glycated hemoglobin in the patients with diabetes was 10.19±2.04%, while in control individuals, this value was estimated to be 4.49±1.07%.The mean values of creatinine and activity of alanine transaminase were higher in diabetic patients (1.18±0.61 mg/dL, 42.94±3.41, respectively) than in healthy controls (0.94±0.27 mg/dL, 36.35±3.09, respectively). The data on the clinical parameters of the male and female study participants in the two groups are presented in Table 2.

### Genotyping of rs3824662 polymorphism in GATA3 gene by allele-specific PCR

**Fig 1:**
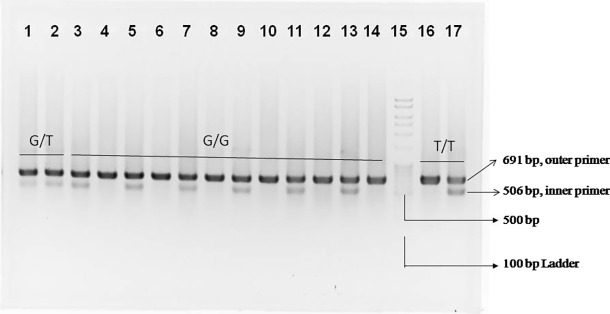
Agarose gel electrophoresis of PCR amplicons of the GATA3 gene containing 691 base pairs amplified with outer primers and 506 base pairs amplified with allele specific primers. Each lane had the common 691 base pair band amplified with outer primer pair. Lanes 1 and 2 indicate the heterozygous genotype (GT), as both inner primers produced PCR products in two different reaction tubes with the same sample. Lanes 3 to 14 indicate the genotype of six samples as homozygous wild type (GG). Lanes 16 and 17 clearly indicate the sample that represents mutant homozygous type (TT), as only the T allele-specific inner primer produced a band of 506 base pairs. Lane 15 indicates the 100 base pair ladder.

The G allele-specific primer bound only with the G nucleotide that is present in the DNA sequence to be amplified, and the T allele-specific primer bound only with T nucleotide. Forward outer and reverse outer primers were used to amplify a 691 base pair sequence of the GATA3 gene. Therefore, the bands containing 691 base pairs were the signature of a positive polymerase chain reaction for the polymorphism study. The allele-specific primer pair was the forward inner primer, which produced a band of 506 base pairs with the reverse outer primer. The reaction mixture contained both forward outer and reverse outer primers and contained either the G allele-specific forward inner primer or the T allele-specific forward inner primer. When the G allele was present in a specific DNA sequence, only the 506 base pair band was found by gel electrophoresis of PCR amplicons with the G allele-specific primer, as shown in lane 3–14 in Fig 1. In this case, no such 506 base pair band was found by gel electrophoresis with the T allele-specific primer, as shown in lanes 3–14 in Fig 1. On the other hand, for mutant TT genotypes, the opposite phenomenon occurred when the T allele was present, which was reflected by the presence of the 506 base pair DNA band only in lane 17, not in lane 16, for the same sample. In the case of the heterozygous genotype (GT), both inner primers for G and T alleles bound to specific DNA sequences as shown in lanes 1–2 as a 506 base pair band.

### Distribution of genotypes and alleles regarding rs3824662 polymorphisms

It was revealed that the distributions of genotypic frequencies (GG, GT, TT) were 51.85%, 39.06%, and 9.09% and 61.94%, 34.41%, and 3.64% in type 2 diabetes patients and control individuals, respectively. For type 2 diabetic patients and control individuals, the frequency distribution of the ancestral G allele was 71.38% and 79.15%, respectively, while for the mutant T allele, it was 28.70% and 20.85%, respectively. Table 3 summarizes the genotypic and allelic distributions of rs3824662 in the GATA3 gene. Statistical analyses revealed that both the mutant allele and genotypes containing the mutant allele were significantly associated with the risk of type 2 diabetes, as shown in Table 3.

**Table 3:** Distribution of genotypic frequencies with respect to the GATA3 gene in Type 2 diabetic patients and healthy individuals.

Parameters		Type 2 diabetic patients n (%)	Healthy Controls n (%)	OR, (95% CI Lowest-Highest)	χ2	p
Genotype	GG/GT/TT	154/116/27	153/85/9	-	-	-
Alleles	G/T	424/170 (71.38/28.62)	391/103 (79.15/20.85)	1.52 (1.15–2.02)	8.66	<0.01
Co-dominant model	GG	154 (51.85)	153 (61.94)	1	-	-
	GT	116 (39.06)	85 (34.41)	2.98 (1.36–6.55)	7.98	<0.01
	TT	27 (9.09)	9 (3.64)	1.36 (0.95–1.94)	2.78	0.095
Dominant model	GG	154	153	1	-	-
	GT+TT	143 (48.14)	94 (38.06)	1.51 (1.07–2.13)	5.33	0.018
Recessive	GG+GT	270 (90.90)	238	1	-	-
	TT	27	9	0.37 (0.17–0.82)	6.47	0.011
Over dominant	GG+TT	181 (60.94%)	162 (65.59)	1	-	-
	GT	116	85	0.82 (0.57–1.16)	1.25	0.263

OR: Odds ratio; CI: Confidence interval; χ2: Chi square

Further, the distributions of genotypic frequencies (GG, GT, TT) were 51.18%, 39.37%, and 9.44% and 61.61%, 34.95%, and 3.88% in male type 2 diabetes patients and control individuals, respectively. For these study participants, the frequency distribution of the ancestral G allele was 70.87% and 78.64%, respectively, while for mutant T allele, it was 29.13% and 21.36%, respectively. Table 4 summarizes the genotypic and allelic distributions of the rs3824662 polymorphism in male type 2 diabetes patients and control individuals. Statistical analysis revealed that the mutant allele of rs3824662 in GATA3 gene was associated with the risk of type 2 diabetes in male participants. However, genotypic frequencies were not found to be associated with disease risk. In female participants, it was found that the distributions of genotypic frequencies (GG, GT, TT) were 52.35%, 38.82%, and 8.82% and 62.5%, 34.03%, and 3.47% in patients with T2D and healthy individuals, respectively. Their respective frequency distribution of the ancestral G allele was 71.76% and 79.51%, while for the mutant T allele, it was 28.24% and 20.49%, respectively. Table 5 summarizes the genotypic and allelic distributions of rs3824662 in the GATA3 gene in female participants. Statistical analyses revealed that both the mutant allele (T) and rare genotype (TT) with the mutant allele were associated with the risk of type 2 diabetes.

**Table 4:** Distribution of genotypic frequencies with respect to the GATA3 gene in male type 2 diabetic patients and healthy individuals.

Parameters		Type 2 diabetic patients n (%)	Healthy Controls n (%)	OR (95% CI Lowest-Highest)	χ2	p
Genotype	GG/GT/TT	65/50/12	63/36/4	-	-	-
Alleles	G/T	180/74 (70.87/29.13)	162/44 (78.64/21.36)	1.51 (0.99–2.33)	3.61	0.05
Co-dominant model	GG	65 (51.18)	63 (61.17)	1	-	-
	GT	50 (39.37)	36 (34.95)	1.35 (0.78–2.34)	1.12	0.29
	TT	12 (9.45)	4 (3.88)	2.91 (0.89–9.50)	3.35	0.07
Dominant model	GG	65	63	1	-	-
	GT+TT	62 (48.82)	40 (38.83)	1.50 (0.89–2.54)	2.28	0.12
Recessive	GG+GT	115 (90.55)	99 (96.12)	1	-	-
	TT	12	4	2.58 (0.81–8.26)	2.72	0.099
Over dominant	GG+TT	77 (60.63)	67 (65.05)	1	-	-
	GT	50	36	1.21 (0.704–2.07)	0.474	0.49

OR: Odds ratio; CI: Confidence interval; χ2: Chi square

**Table 5:** Distribution of genotypic frequencies with respect to the GATA3 gene in female Type 2 diabetic patients and healthy individuals.

Parameters		Type 2 diabetic patients n (%)	Healthy Controls n (%)	OR (95% CI Lowest-Highest)	χ2	p
Genotype	GG/GT/TT	89/66/15	90/49/5	-	-	-
Alleles	G/T	244/96 (71.76/28.24)	229/59 (79.51/20.49)	1.53 (1.05–2.21)	5.04	0.025
Co-dominant model	GG	89 (52.35)	90 (62.5)	1	-	-
	GT	66 (38.82)	49 (34.03)	1.36 (0.85–2.18)	1.65	0.2
	TT	15 (8.82)	5 (3.47)	3.03 (1.06–8.70)	4.61	0.031
Dominant model	GG	89	90	1	-	-
	GT+TT	81 (47.65)	54 (37.5)	1.51 (0.96–2.38)	3.27	0.07
Recessive	GG+GT	155 (91.18)	139 (96.53)	1	-	-
	TT	15	5	2.69 (0.95–7.59)	3.74	0.053
Over dominant	GG+TT	104 (61.18)	95 (65.97)	1	-	-
	GT	66	49	1.23 (0.774–1.95)	0.772	0.379

OR: Odds ratio; CI: Confidence interval; χ2: Chi square

### Evaluation of the amplified PCR products of the STAT4 gene

**Fig 2:**
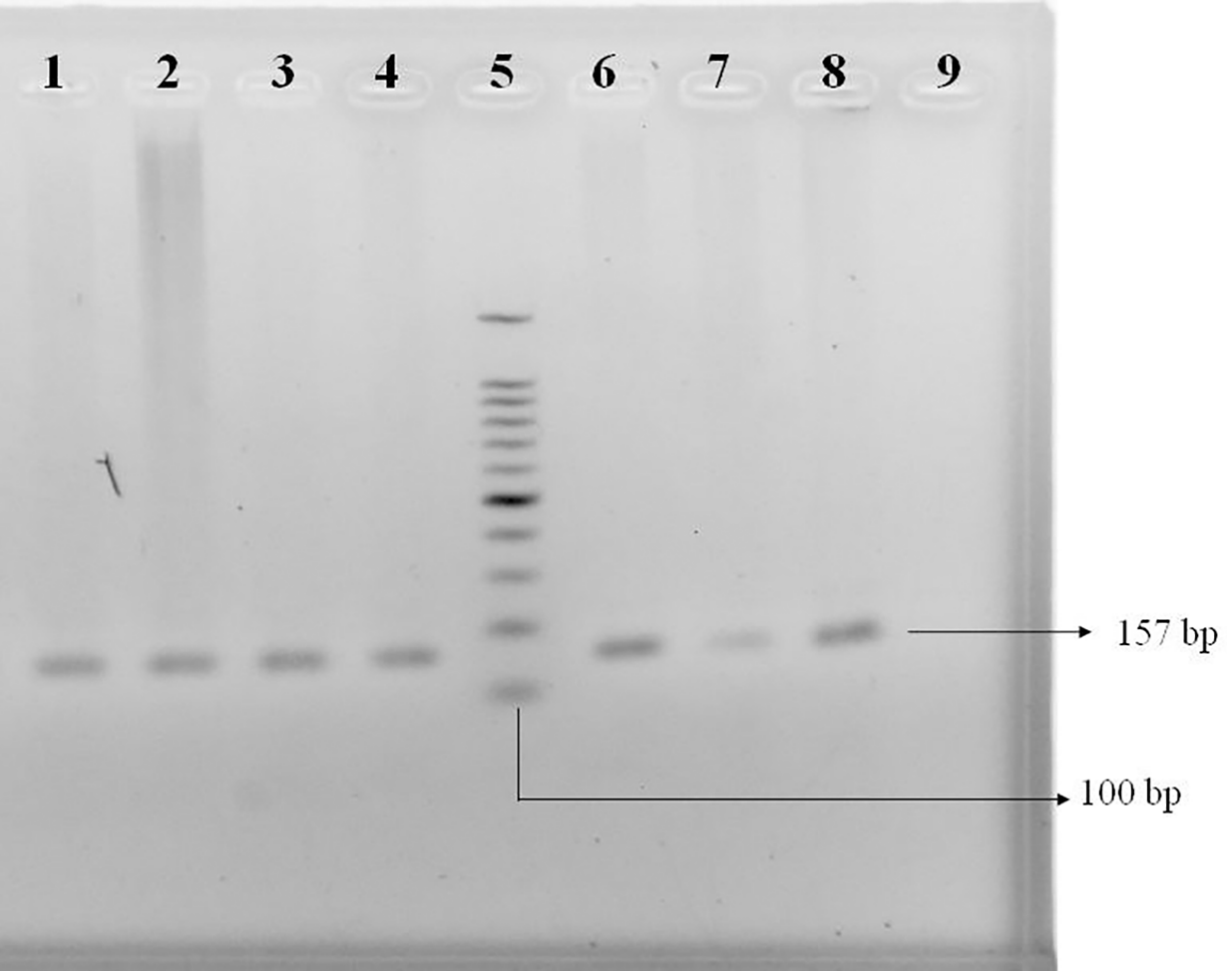
Agarose gel electrophoresis of PCR amplicons amplified for the STAT4 gene comprising 157 base pairs. Lanes 1, 2, 3, 4, 6, 7, and 8 designate PCR products of template DNA from study participants, and lane 9 designates a negative control in which no DNA was used as a template. Lane 5 indicates a 100 base pair DNA ladder.

To confirm the sizes of the PCR amplicons of the STAT4 gene from healthy individuals and T2D patients, a 100 base pair DNA ladder (Cleaver Scientific Ltd, UK) was used. Lane NC represents the negative control, which was subjected to polymerase chain reaction without template DNA. Different DNA bands are presented in Fig 2.

**Fig 3:**
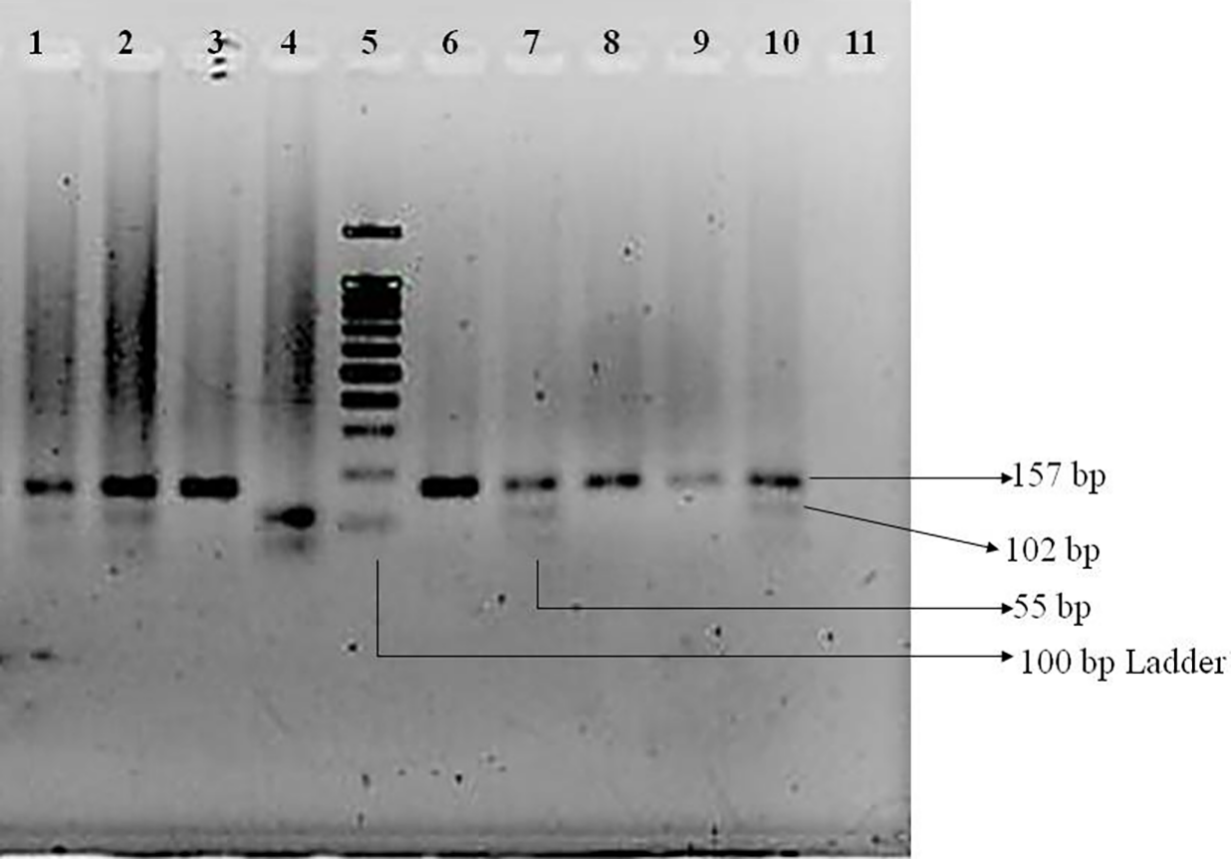
Agarose gel electrophoresis of *DdeI* digested products of PCR amplicons of STAT4. Lane 11 contains the negative control. Here, DNA fragments of 157, 102, and 55 base pairs were found. Lanes 3, 6, 8, and 9 represent undigested bands of 157 base pairs, which represent the wild-type CC homozygote. Lanes 1, 2, 7, and 10 represent bands of 157, 102, and 55 base pairs that clearly indicate the presence of the heterozygous CG allele. Lane 4 represents completely digested bands of 102 and 55 base pairs that indicate the mutant GG allele. Lane 5 indicates the 100 base pair ladder.

The presence of the rs10181656 polymorphism in the STAT4 gene was determined after digesting 157 base pair PCR products with *Dde*I (C↓TNAG) (Thermo Fisher Scientific, USA) restriction enzyme followed by 2.5% agarose gel electrophoresis and ethidium bromide staining, as shown in Fig 3. The *Dde*I cleaved the 157 base pair PCR products into two fragments of 102 base pairs and 55 base pairs when the G allele was present, whereas the C allele was not digested and displayed only the 157 base pair band. In the case of heterozygous GC, 157 base pair, 102 base pair and 55 base pair bands were found.

### Distribution of genotypes and alleles regarding rs10181656 polymorphisms

It was revealed that the distributions of genotypic frequencies (CC, CG, GG) were 60.27%, 29.63%, and 10.10% and 57.49%, 33.19%, and 9.31% in type 2 diabetic patients and control individuals, respectively. For type 2 diabetic patients and control individuals, the frequency distribution of the ancestral C allele was 75.09% and 74.09%, respectively, while for mutant G allele, it was 24.91% and 25.91%, respectively. Table 6 summarizes the genotypic and allelic distributions of rs10181656 in the STAT4 gene. Statistical analyses revealed that neither allelic nor genotypic variations showed an association with the risk of type 2 diabetes.

**Table 6:** Distribution of genotypic frequencies with respect to the STAT4 gene in Type 2 diabetic patients and healthy individuals.

Parameters		Type 2 diabetic patients n (%)	Healthy Controls n (%)	OR (95% CI Lowest-Highest)	χ2	p
Genotype	CC/CG/GG	179/88/30	142/82/23	-	-	-
Alleles	C/G	446/148 (75.09/24.91)	366/128 (74.09/25.91)	0.948 (0.721–1.247)	0.141	0.707
Co-dominant model	CC	179 (60.27)	142 (57.49)	1	-	-
	CG	88 (29.63)	82 (33.19)	0.85 (0.59–1.24)	0.716	0.397
	GG	30 (10.10)	23 (9.31)	1.03 (0.58–1.86)	0.013	0.91
Dominant model	CC	179	142	1	-	-
	CG+GG	118 (39.73)	105 (42.51)	0.89 (0.63–1.26)	0.431	0.51
Recessive	CC+CG	267 (89.90)	224 (90.69)	1		-
	GG	30	23	1.09 (0.62–1.94)	0.095	0.76
Over dominant	CC+GG	209 (70.37%)	165 (66.80)	1	-	-
	CG	88	82	0.85(0.59–1.22)	0.799	0.37

OR: Odds ratio; CI: Confidence interval; χ2: Chi square

Further, the distributions of genotypic frequencies (CC, CG, GG) were 62.12%, 28.79%, and 9.09% and 57.27%, 32.73%, and 10.0% in male type 2 diabetic patients and control individuals, respectively. For these participants, the frequency distribution of the ancestral C allele was 76.52% and 73.64%, respectively, while for the mutant G allele, it was 23.48% and 26.36%, respectively. Table 7 summarizes the genotypic and allelic distributions with respect to the rs3824662 polymorphism in male type 2 diabetic patients and control individuals. Allelic and genotypic frequencies were not found to be associated with the risk of type 2 diabetes in male participants. In the case of female participants, it was found that the distributions of genotypic frequencies (CC, CG, GG) were 58.79%, 30.30%, and 10.91% and 57.66%, 31.39%, and 10.95% in patients with type 2 diabetes and healthy individuals, respectively. Their respective frequency distribution of the ancestral C allele was 73.94% and 73.36%, while for the mutant G allele, it was 26.06% and 26.64%, respectively. Table 8 summarizes the genotypic and allelic distributions of rs10181656 in the STAT4 gene of female participants. Neither allelic nor genotypic variation was found to be associated with the risk of type 2 diabetes in female participants.

**Table 7:** Distribution of genotypic frequencies with respect to the STAT4 gene in male Type 2 diabetic patients and healthy individuals.

Parameters		Type 2 diabetic patients n (%)	Healthy Controls n (%)	OR (95% CI Lowest-Highest)	χ2	p
Genotype	CC/CG/GG	82/38/12	63/36/11	-	-	-
Alleles	C/G	202/62 (76.52/23.48)	162/58 (73.64/26.36)	0.857 (0.566–1.296)	0.533	0.465
Co-dominant model	CC	82 (62.12)	63 (57.27)	1	-	-
	CG	38 (28.79)	36 (32.73)	0.81 (0.46–1.42)	0.535	0.46
	GG	12 (9.09)	11 (10.00)	0.84 (0.35–2.02)	0.154	0.69
Dominant model	CC	82	63	1		
	CG+GG	50 (37.88)	47 (42.73)	0.82 (0.49–1.37)	0.587	0.44
Recessive	CC+CG	120 (90.91)	99 (90.0)	1		
	GG	12	11	0.90 (0.38–2.13)	0.057	0.81
Over dominant	CC+GG	94 (71.21)	74 (67.27)	1	-	-
	CG	38	36	0.83 (0.48–1.44)	0.438	0.51

OR: Odds ratio; CI: Confidence interval; χ2: Chi square

**Table 8:** Distribution of genotypic frequencies with respect to the STAT4 gene in female Type 2 diabetic patients and healthy individuals.

Parameters		Type 2 diabetic patients n (%)	Healthy Controls n (%)	OR (95% CI Lowest-Highest)	χ2	p
Genotype	CC/CG/GG	97/50/18	79/43/15	-	-	-
Alleles	C/G	244/86 (73.94/26.06)	201/73 (73.36/26.64)	0.97 (0.674–1.395)	0.026	0.87
Co-dominant model	CC	97 (58.79)	79 (57.66)	1		
	CG	50 (30.30)	43 (31.39)	0.95 (0.57–1.57)	0.044	0.83
	GG	18 (10.91)	15 (10.95)	0.98 (0.46–2.06)	0.003	0.95
Dominant model	CC	97	79	1		
	CG+GG	68 (41.21)	58 (42.34)	0.95 (0.60–1.51)	0.038	0.84
Recessive	CC+CG	147 (89.09)	122 (89.05)	1		
	GG	18	15	0.99 (0.48–2.06)	0.0001	0.99
Over dominant	CC+GG	115 (69.70)	94 (68.61)	1		
	CG	50	43	0.95 (0.58–1.55)	0.041	0.84

OR: Odds ratio; CI: Confidence interval; χ2: Chi square

## Discussion

Diabetes mellitus is a complex polygenic metabolic disorder that is caused by environmental and genetic factors. Prevalence rates differ across ethnicities, with the prevalence in the Bangladeshi population projected to reach 13% by the year 2030. In Bangladesh, almost 60% of the total adult population is under the age of 40 years. With the growing economy of the country, the health of the abundant workforce should be addressed. According to the World Health Organization, non-communicable diseases (NCDs) are silent killers that cause 70% of all deaths in the world, corresponding to 40 million people per year, with people from developing and low- and low-middle-income countries suffering the most. Among the NCDs, diabetes is the 4^th^ largest cause of morbidity and mortality in the world population [34].

Non-genetic environmental factors have been attributed to the onset of diabetes in different ethnic groups. However, even living in the same environment, different ethnic groups have different prevalence rates of diabetes, which is evidence of an association of genetic factors with diabetes [35]. Different risk alleles or SNPs, either independently or cumulatively, have shown their association with the incidence of diabetes in different populations including in the Asians [36–40] Moreover, intronic variants, the hidden treasures within our genome, have been identified to be associated with different inherited diseases with varying degrees of risk. It is now well scripted that mutations in the exon-intron splice junctions are associated with the pathogenicity of diseases and accounted for ~10% of genetic mutations (HGMD®; http://www.hgmd.org) [41]. A meta-analysis of genome-wide association data revealed relationship of six new loci in European descent with the pathogenesis of type 2 diabetes while another study identified association of 18 SNPs that represented only 6% proportion of heritable linkage [42,43]. Intronic regions contain functional polymorphisms along with pathological mutations. Although multiple genetic risk variants associated with type 2 diabetes have been identified in different populations [10,44,45], evidence for an association of SNPs in the Bangladeshi population is very inadequate. Thus, in the present study, an effort is made to determine the association between T2D risk and SNPs present in GATA3 and STAT4 transcription factors.

When total study participants were considered, out of three genotypes, only the heterozygous variant (rs3824662 GT) independently had a significant association with the risk of disease [odds ratio 2.98 (95% CI 1.36–6.55), *p*<0.01], while the over-dominant model had no effect. Even when either one allele or both alleles were mutated, the study participants were at risk of type 2 diabetes, which is reflected in the dominant model (comparing GG vs GT+TT) [odds ratio 1.51 (95% CI 1.07–2.13), *p* = 0.018] or recessive model (comparing GG+TT vs TT) [odds ratio 0.37 (95% CI 0.17–0.82), *p* = 0.011] (Table 3). When study participants were grouped according to their gender, the rs3824662 T allele was associated with the risk of type 2 diabetes in both male and female patients (Tables 4 and 5). However, female participants showed an association with the risk of T2D when co-dominant and dominant models were considered, while none of the models showed any association with disease risk in male participants. Moreover, though mutant genotype rs3824662 TT inclined towards its association with the risk of type 2 diabetes only in female participants, however, a definitive remark can only be drawn from a study with large and almost equal number of male and female participants. The mechanism by which the mutant T allele in GATA3 is associated with the risk of diseases is still unclear. However, by analyzing the SNP rs3824662 using a 3DSNP database, it was revealed that this SNP is associated with 20 different SNPs identified within intronic sequences of GATA binding protein 3 (S1 Table). 3DSNP comprehensively annotates the regulatory function of human non-coding SNPs by investigating three-dimensional interactions with genes and genetically associated SNPs mediated by chromatin loops. According to functionality scores, it was further observed that rs3824662 is involved in modulating regulatory motifs such as enhancers, transcription factor binding motifs, promoter regions (S2 Table). Thus, due to association with a large number of SNPs and regulatory motifs, rs3824662 may alter the expression pattern of the GATA3 gene, which in turn may affect regulatory T cells, followed by onset of type 2 diabetes through developing insulin resistance.

Although diabetes mellitus has been broadly classified as type 1 and type 2, increasing evidence has shed light on the fact that these two diseases overlap in many aspects. For example, classical immunological parameters for type 1 diabetes, such as anti-islet cell antibodies, elevated circulating cytokines and chemokines, are also present in many patients with type 2 diabetes [46,47]. In addition, obesity, which is associated with insulin resistance and type 2 diabetes, shows strong correlations with the increased incidence of type 1 diabetes [48,49]. Signal transducer and activator of transcription 4 (STAT4) is a transcription factor that is involved in modulating immune functions by transducing signals as a result of interaction between cytokines/chemokines and receptors. STAT4 is activated by IL-12 and has been found to be associated with both type 1 and type 2 diabetes. Protection of STAT4 activation inhibits the development of autoimmune diabetes or type 1 diabetes in non-obese diabetic mice [30,50]. As a transcription factor, STAT4 has appeared to be a major regulator of T-cell activation, macrophage inflammatory phenotype, insulin resistance and atherosclerosis [51,52]. Further, STAT4 mediates inflammatory responses in immune cells and adipocytes in diabetes and obesity. STAT4 also has a role in skin wounds, as demonstrated in a typical type 2 diabetic mouse model [53].

The STAT4 gene polymorphism is associated with increased risk for the development of early onset type 1 diabetes [30] but not for type 2 diabetes on the island of Crete, a well-defined area with a genetically homogeneous population [54]. A meta-analysis study reported a significant association of the STAT4 rs7574865 polymorphism with the risk of type 1 diabetes [55]. It was further revealed that both Asians and Caucasians with the STAT4 rs7574865 polymorphism have an increased diabetes risk. Although involvement of STAT4 activation has been an overlapping clinical feature for both type 1 diabetes and type 2 diabetes, the role of rs10181656 in STAT4 is not yet clear and remains to be elucidated in the Bangladeshi population. SNP rs7574865 is located within intron 3 of a noncoding region of STAT4. STAT4, through its interaction with IL-12, plays pivotal role in TH¬1 immune responses and IFN-γ transcription. It is suspected that rs7574865 may influence the gene expression of STAT4 at the level of transcription and variant splicing [56], or it may be linked to causative mutations. Interestingly, a 3DSNP database revealed that rs10181656 is in linkage disequilibrium with 17 other SNPs present in the intronic sequences of STAT4 (S3 Table) and causes change in motifs (S4 Table). Most importantly, our SNP of interest has been found to be associated with the extensively studied, type 1 diabetes-associated STAT4 SNP rs7574865. Genetic variants of STAT4 may be involved in regulating the balance of IL-12 versus IL-23 effects and may affect the prevalence of inflammatory diseases via dysregulation of TH1 and TH17 differentiation. However, allelic and genotypic variations were not found to be associated with the risk of type 2 diabetes in study participants. Moreover, none of the co-dominant, dominant, recessive, or over-dominant models of the STAT4 rs10181656 polymorphism in the total study sample or in the male or female participants with type 2 diabetes showed an association with the risk of disease (Tables 6, 7 and 8).

Our findings demonstrate that genetic variation of the transcription factor GATA3, not STAT4, is associated with the risk of type 2 diabetes in the Bangladeshi population. The mechanism behind these roles needs to be investigated further. Moreover, studies with a large number of study participants from different ethnic populations are warranted to confirm the findings of this study.

## Supporting information

S1 Table GATA3 intronic variant showing association with several SNPs that are tagged with diseases in different population of the world. (DOC)

S2 Table Association of rs3824662 with different SNPs that have effects on enhancers, promoters and transcription factor binding (TFBS) proteins obtained from 3DSNP database. (DOC)

S3 Table STAT4 intronic variant showing association with several SNPs that are tagged with diseases in different population of the world. (DOC)

S4 Table Association of rs10181656 with different SNPs that have effects on enhancers and transcription factor binding (TFBS) proteins obtained from 3DSNP database. (DOC)

S1 Data Huda et al- All raw data combined cases controls. This excel file contains the raw data for this study.
(XLS)
