# Supplementary material for: Genetic variation of the transcription factor GATA3, not STAT4, is associated with the risk of type 2 diabetes in the Bangladeshi population
Source: PLoS One. 2018 Jul 25;13(7):e0198507. doi: 10.1371/journal.pone.0198507 (PMC6059405; doi:10.1371/journal.pone.0198507)
Supplement: S1 Table — (DOC) [file pone.0198507.s001.doc]

**S1 Table. GATA3 intronic variant showing association with several SNPs that are tagged with diseases in different population of the world.**

| **SNP of interest** | **Associated SNP** | **R-squared** | **D'** | **Population** | **Nucleotides** | **Ancestral** | **Variants** |
| --- | --- | --- | --- | --- | --- | --- | --- |
| rs3824662 | rs477771 | 0.87 | 1 | EAS | G/T | G | intron variant |
|  | rs444762 | 0.93 | 1 | EAS | A/C | C |  |
|  | rs376397 | 0.93 | 1 | EAS | A/G | G |  |
|  | rs371668 | 0.86 | 1 | EAS | C/T | T |  |
|  | rs3824661 | 0.86 | 1 | EAS | A/T | A |  |
|  | rs11255504 | 0.93 | 0.97 | AMR | A/T | A |  |
|  |  | 0.9 | 0.98 | EAS |  |  |  |
|  |  | 0.9 | 0.99 | EUR |  |  |  |
|  |  | 0.93 | 0.97 | SAS |  |  |  |
|  | rs409560 | 0.93 | 1 | EAS | C/G | C |  |
|  | rs3781092 | 0.82 | 0.99 | EAS | A/C/T | C |  |
|  |  | 0.81 | 0.95 | EUR |  |  |  |
|  |  | 0.93 | 0.97 | SAS |  |  |  |
|  | rs3781093 | 0.88 | 0.97 | AMR | A/G | A |  |
|  |  | 0.93 | 0.99 | EAS |  |  |  |
|  |  | 0.87 | 0.98 | EUR |  |  |  |
|  |  | 0.93 | 0.98 | SAS |  |  |  |
|  | rs374641 | 0.86 | 1 | EAS | C/T | C |  |
|  | rs386680 | 0.86 | 1 | EAS | C/G/T | G |  |
|  | rs7094966 | 0.8 | 0.98 | EAS | G/T | T |  |
|  |  | 0.93 | 0.98 | SAS |  |  |  |
|  | rs2280015 | 0.88 | 0.96 | SAS | A/C/G | G |  |
|  | rs3802600 | 0.8 | 0.98 | EAS | A/T | T |  |
|  |  | 0.93 | 0.98 | SAS |  |  |  |
|  | rs11567927 | 0.87 | 0.96 | SAS | -/A | G |  |
|  | rs11567923 | 0.82 | 0.96 | SAS | -/T | C |  |
|  | rs10905282 | 0.88 | 0.96 | SAS | G/T | T |  |
|  | rs10905281 | 0.88 | 0.94 | SAS | A/C/T | C |  |
|  | rs11255507 | 0.88 | 0.96 | SAS | G/T | G |  |
|  | rs10905280 | 0.93 | 0.98 | SAS | C/T | C |  |

EAS: East Asians; SAS: South Asians; EUR: Europeans; AMR: Americans
