# Supplementary material for: Genetic variation of the transcription factor GATA3, not STAT4, is associated with the risk of type 2 diabetes in the Bangladeshi population
Source: PLoS One. 2018 Jul 25;13(7):e0198507. doi: 10.1371/journal.pone.0198507 (PMC6059405; doi:10.1371/journal.pone.0198507)
Supplement: S2 Table — (DOC) [file pone.0198507.s002.doc]

**S2 Table. Association of rs3824662 with different SNPs that have effects on enhancers, promoters and transcription factor binding (TFBS) proteins obtained from 3DSNP database.**

| SNP | Position | Ref/Alt | Score | Linear closest gene | Enhancer | Promoter | TFBS | Motif |
| --- | --- | --- | --- | --- | --- | --- | --- | --- |
| rs3824662 | chr10:8104207 | C/A | 33.55 | GATA3 | 47 | 1 | 6 | 0 |
