# Supplementary material for: Genetic variation of the transcription factor GATA3, not STAT4, is associated with the risk of type 2 diabetes in the Bangladeshi population
Source: PLoS One. 2018 Jul 25;13(7):e0198507. doi: 10.1371/journal.pone.0198507 (PMC6059405; doi:10.1371/journal.pone.0198507)
Supplement: S3 Table — (DOC) [file pone.0198507.s003.doc]

**S3 Table. STAT4 intronic variant showing association with several SNPs that are tagged with diseases in different population of the world.**

| **SNP of interest** | **Associated SNP** | **R-squared** | **D’** | **Population** | **Nucleotides** | **Ancestral** | **Variants** |
| --- | --- | --- | --- | --- | --- | --- | --- |
| rs10181656 | rs11889341 | 0.97 | 0.99 | AMR | C/T | C | intron variant |
|  |  | 0.84 | 0.94 | EAS |  |  |  |
|  |  | 0.97 | 1 | EUR |  |  |  |
|  | rs10168266 | 0.8 | 0.91 | AMR | C/T | C |  |
|  | rs10174238 | 0.85 | 0.99 | AMR | A/G | A |  |
|  |  | 0.89 | 0.97 | EUR |  |  |  |
|  | rs12612769 | 0.9 | 0.99 | AMR | A/C/T | A |  |
|  |  | 0.86 | 0.98 | EAS |  |  |  |
|  | rs4274624 | 0.99 | 1 | AMR | C/T | T |  |
|  |  | 0.98 | 0.99 | EAS |  |  |  |
|  |  | 0.99 | 1 | EUR |  |  |  |
|  |  | 0.99 | 1 | SAS |  |  |  |
|  | rs7582694 | 0.98 | 1 | AMR | C/G | C |  |
|  |  | 0.99 | 1 | EAS |  |  |  |
|  |  | 1 | 1 | EUR |  |  |  |
|  |  | 0.97 | 1 | SAS |  |  |  |
|  | rs7574865 | 0.98 | 0.99 | AMR | G/T | T |  |
|  |  | 0.98 | 1 | EAS |  |  |  |
|  |  | 0.98 | 1 | EUR |  |  |  |
|  |  | 0.99 | 1 | SAS |  |  |  |
|  | rs71030323 | 0.83 | 0.99 | AMR | -/A | G |  |
|  |  | 0.83 | 0.99 | EAS |  |  |  |
|  |  | 0.86 | 0.98 | EUR |  |  |  |
|  | rs7568275 | 0.96 | 0.99 | AMR | C/G | G |  |
|  |  | 0.97 | 0.99 | EAS |  |  |  |
|  |  | 0.99 | 1 | EUR |  |  |  |
|  |  | 1 | 1 | SAS |  |  |  |
|  | rs10553577 | 0.96 | 0.98 | AMR | -/ATA | N/A |  |
|  | rs8179673 | 1 | 1 | AMR | C/T | T |  |
|  | rs10553577 | 0.91 | 0.98 | EAS | -/ATA | N/A |  |
|  | rs8179673 | 0.99 | 1 | EAS | C/T | T |  |
|  | rs10553577 | 0.94 | 0.97 | EUR | -/ATA | N/A |  |
|  | rs8179673 | 0.99 | 1 | EUR |  | T |  |
|  |  | 1 | 1 | SAS | C/T |  |  |
|  | rs4853458 | 0.99 | 1 | AMR | A/G | G |  |
|  |  | 0.97 | 0.99 | EAS |  |  |  |
|  |  | 0.98 | 1 | EUR |  |  |  |
|  |  | 0.99 | 1 | SAS |  |  |  |
|  | rs4853459 | 0.87 | 0.99 | AMR | C/T | T |  |
|  |  | 0.85 | 0.99 | EAS |  |  |  |
|  |  | 0.95 | 0.99 | EUR |  |  |  |
|  |  | 0.86 | 0.99 | SAS |  |  |  |

EAS: East Asians; SAS: South Asians; EUR: Europeans; AMR: Americans
