# Supplementary material for: Genetic variation of the transcription factor GATA3, not STAT4, is associated with the risk of type 2 diabetes in the Bangladeshi population
Source: PLoS One. 2018 Jul 25;13(7):e0198507. doi: 10.1371/journal.pone.0198507 (PMC6059405; doi:10.1371/journal.pone.0198507)
Supplement: S4 Table — (DOC) [file pone.0198507.s004.doc]

**S4 Table. Association of rs10181656 with different SNPs that have effects on enhancers and transcription factor binding (TFBS) proteins obtained from 3DSNP database.**

| SNP | Position | Ref/Alt | Linear closest gene | Enhancer | Promoter | TFBS | Motif |
| --- | --- | --- | --- | --- | --- | --- | --- |
| rs10181656 | chr2:191969878 | G/C | STAT4 | 4 | 0 | 1 | 0 |
